# Fluorosis dental en zonas volcánicas: análisis de factores asociados en una población escolar colombiana

**DOI:** 10.7705/biomedica.7337

**Published:** 2025-05-30

**Authors:** Yeimy Tatiana Ortega, Angye Paola Salcedo, Rafaela Reis da Silva, Bruno Gutiérrez, Johana Alejandra Moreno

**Affiliations:** 1 Salud Pública Bucodental, Escuela de Odontología, Grupo de Investigación Pacífico Siglo XXI, Universidad del Valle, Cali, Colombia Universidad del Valle Salud Pública Bucodental Escuela de Odontología Universidad del Valle Cali Colombia; 2 Faculdade de Odontologia, Universidade Federal de Minas Gerais, Belo Horizonte, Brasil Universidade Federal de Minas Gerais Faculdade de Odontologia Universidade Federal de Minas Gerais Belo Horizonte Brazil

**Keywords:** fluorosis dental, niño, estudiantes, flúor., fluorosis, dental, child, students, fluorine

## Abstract

**Introducción.:**

La fluorosis dental es un problema endémico en el sur de Colombia, especialmente por su cercanía a zonas volcánicas y la composición de sus aguas.

**Objetivo.:**

Identificar la prevalencia y los factores asociados con el desarrollo de fluorosis dental en la población escolar del municipio de La Cruz (Nariño).

**Materiales y métodos.:**

Se realizó un estudio observacional de corte transversal, en el que se incluyeron variables clínicas y sociodemográficas, y datos sobre hábitos, dieta y conocimiento sobre fluorosis dental. Para la recolección de la información se usó un cuestionario que fue diligenciado por los padres o tutores y se llevó a cabo una evaluación clínica -previamente estandarizada por los investigadores- de estudiantes entre los 8 y los 12 años de dos instituciones educativas.

Se hizo un análisis estadístico descriptivo con proporciones y medidas de tendencia central. Para el análisis multivariado, se utilizó una regresión binomial negativa con razones de tasa e intervalos de confianza del 95 %.

**Resultados.:**

Los 116 participantes tuvieron una edad media de 9,80 (DE = 1,38) años y una media del índice de Dean de 2,36 (DE = 0,86). Se evidenció un mayor índice de Dean a mayor edad (p < 0,003), marca de sal (p < 0,007), mayor consumo de legumbres (p < 0,001), cereales (p < 0,038) y pescado (p < 0,025), y menor consumo de carnes rojas (p < 0,017).

**Conclusión.:**

La fluorosis dental es un problema multifactorial que requiere diferentes abordajes según las particularidades de cada territorio, así como enfoques integrales de salud pública para controlar sus altos valores de prevalencia.

## Abstract (en)

**Introduction.:**

Dental fluorosis is an endemic problem in southern Colombia, especially due to its proximity to volcanic areas and the composition of their waters.

**Objective.:**

To identify the prevalence and factors associated with the development of dental fluorosis in the school population of the municipality of La Cruz, Nariño.

**Materials and methods.:**

We conducted a cross-sectional observational study that included clinical and sociodemographic variables and data about habits, diet, and dental fluorosis knowledge. To collect the information, we used a questionnaire completed by the parents or guardians and a clinical evaluation -previously standardized by the researchers- of schoolchildren between 8 and 12 years from two educational institutions.

A descriptive statistical analysis was performed with proportions and measures of central tendency. The multivariate analysis used a negative binomial regression with rate ratios and 95% confidence intervals.

**Results.:**

We included 116 participants with a mean age of 9.80 (SD = 1.38) years and a mean of the Dean’s index of 2.36 (SD = 0.86). A higher Dean’s index was associated with older age (p < 0.003), brand of salt (p < 0.007), higher consumption of legumes (p < 0.001), cereals (p < 0.038), and fish (p < 0.025), and lower consumption of red meat (p < 0.017).

**Conclusion.:**

Dental fluorosis is a multifactorial problem that requires different approaches according to the territories and their particularities and public health measures to control its high prevalence.

La fluorosis dental es una enfermedad de salud pública, endémica en muchas partes del mundo, caracterizada por la hipomineralización del esmalte ^1^. Esta afección se produce como respuesta a la ingestión de flúor por un periodo largo durante la formación del esmalte ^2^. Clínicamente, se manifiesta como manchas, líneas o estrías blancas opacas; en la fluorosis moderada o grave produce manchas marrones ^3^. La fluorosis es una alteración específica durante la formación de los dientes y una condición estética crónica -inducida por fluoruro- en la que se interrumpe el desarrollo del esmalte en formación, haciéndolo más poroso. El grado y la extensión dependen de la concentración de fluoruro en los fluidos tisulares durante el desarrollo de los dientes ^4^. La fase de maduración temprana del desarrollo dental es el período más crítico para el desarrollo de la fluorosis ^5^.

En los países en desarrollo, el estado nutricional y el tipo de dieta están asociados con un mayor riesgo de fluorosis. No obstante, también existen comunidades cuya agua potable contiene una gran concentración de fluoruro ^6^. El consumo de ciertos alimentos ricos en fluoruro o cultivados en áreas con grandes concentraciones de este compuesto en el agua para riego, así como el uso y consumo de productos de higiene oral como pasta dental, pueden afectar las condiciones de salud y desempeñar un papel importante en el desarrollo de la fluorosis ^7^.

En el contexto mundial, la prevalencia de fluorosis es del 7,7 al 80,7 % en áreas con agua fluorada, mientras que, en áreas con presencia de flúor proveniente de otras fuentes, es del 2,9 al 42%; la presentación clínica leve es la más frecuente ^8^. De acuerdo con los últimos estudios realizados en Colombia y las Encuestas Nacionales de Salud Bucal (ENSAB) más recientes, entre el 1998 y el 2014, la prevalencia de fluorosis se ha incrementado, pasó del 11,5 % en 1998 en -personas de 6, 7, 12 y 19 años- al 8,43 %, 62,15 % y 56,05 % en edades de 5, 12 y 15 años, respectivamente. La última encuesta del 2014 evidenció que la prevalencia es mayor en zonas rurales dispersas (64,87 %) y en la región Pacífica (78 %) ^9^. A nivel nacional, durante el 2018, las lesiones clasificadas como “muy leve” alcanzaron la mayor proporción (38,4 %). Sin embargo, los casos con fluorosis “grave”, se localizaron en los departamentos de Caldas (4,1 %), Sucre (2,8 %), Antioquia, Nariño y Casanare (2,6 %) ^10^.

En cuanto a la cuantificación de fluoruro en el agua, en el departamento de Nariño se reportaron concentraciones superiores a 4 ppm. Nariño fue el departamento con mayor número de municipios en riesgo grave (Iles, Ricaurte, San Pedro de Cartago, Taminango, Potosí, Sandoná y Olaya Herrera). Los municipios de La Cruz, La Unión, Olaya Herrera, Imués y San Pablo tenían concentraciones de fluoruro entre 1,01 y 4 ppm, que situaron a La Cruz en riesgo medio para fluorosis dental ^9^. Cerca al municipio de La Cruz, se localiza el complejo volcánico Doña Juana (a 4.200 m), Ánimas (a 4.300 m) y Petacas (a 4.500 m). Esta cercanía podría incidir en que los fluoruros liberados por el volcán sean transportados por el viento y la lluvia a los suelos y aguas circundantes, lo que podría explicar la gran concentración de fluoruro en el agua para consumo humano (entre 1,01 y 4 ppm) ^11^. Los volcanes son una fuente natural de flúor, pero también, son la principal fuente de abastecimiento de agua potable para el consumo humano y animal ^12^.

El objetivo del presente estudio fue identificar la prevalencia de fluorosis dental y los factores asociados con su desarrollo en la población escolar de dos instituciones del municipio de La Cruz (Nariño).

## Materiales y métodos

Este estudio observacional y de corte transversal cuenta con el aval número 005-023 del Comité de Ética en Investigación en Salud de la Universidad del Valle.

El tamaño de la muestra se obtuvo considerando una prevalencia de fluorosis leve del 12 % ^13^, con un intervalo de confianza del 95 %, y una población escolar de 396 estudiantes entre los 8 y los 12 años, pertenecientes a la Institución Educativa Escuela Normal Superior del Mayo y a la Institución Educativa Técnica San Francisco de Asís del municipio de La Cruz (Nariño).

Los criterios de inclusión fueron: rango de edad entre los 8 y los 12 años, haber residido toda la vida en el municipio de La Cruz y tener el aval de participación de los padres mediante la firma del consentimiento informado. Se incluyeron escolares que no utilizaban aparatología fija (ortodoncia, ortopedia), con dentición mixta y permanente.

Los criterios de exclusión fueron escolares con dientes fracturados en más del 50 % de la superficie, lesiones cariosas que impidieran el correcto diagnóstico de la fluorosis dental, lesiones dentales o articulares que obstaculizaran el examen intraoral (por ejemplo, úlceras, abrasión dental, erosión, abfracción, atrición, laceraciones o limitación de la apertura mandibular).

Los participantes fueron seleccionados aleatoriamente en los grados segundo a sexto. Antes de la valoración, se diligenciaron el asentimiento y el consentimiento informado.

### Examen clínico

Se hizo una estandarización de la evaluación clínica entre los examinadores y, como resultado, se obtuvo un coeficiente kappa de Cohen de 0,740, que indicó una concordancia satisfactoria. Posteriormente, se hizo un estudio piloto para analizar las preguntas del cuestionario, comprobar si los pacientes entendían los instrumentos y hacer las correcciones necesarias.

Los pacientes fueron examinados durante 15 minutos aproximadamente, no sin antes eliminar -mediante cepillado- residuos alimenticios, placa o alguna sustancia que interfiriera con la visualización de los dientes. Las observaciones se registraron en la tabla epidemiológica del índice de Dean (material suplementario).

Se analizó la presencia o ausencia de fluorosis dental en cada una de las superficies vestibulares y se determinó su gravedad. El grado de compromiso por la fluorosis se calificó de 0 a 5, como sigue ^14^:

0: normal; la superficie del esmalte es lisa, brillante y, generalmente, de un color blanco crema pálido;
1: cuestionable o dudosa; el esmalte muestra ligeras aberraciones respecto a su translucidez en un diente sano; estas fluctúan entre unas pocas manchas blancas y manchas ocasionales;
2: muy leve; hay pequeñas zonas opacas, de color blanco papel, diseminadas irregularmente en menos del 25 % de la superficie dental vestibular;
3: leve; hay zonas opacas blancas extendidas entre el 25 % y menos del 50 % de la superficie dental vestibular;
4: moderada; las superficies del esmalte dental muestran un marcado desgaste y manchas carmelitas o marrones que, a menudo, se asocian con una característica desfigurante, y
5: grave; las superficies del esmalte están muy afectadas y la hipoplasia es tan importante que la forma general del diente se puede ver afectada. Existen fosas discontinuas o confluyentes, las manchas marrones están extendidas y los dientes tienen un aspecto erosionado o una apariencia de corrosión.

### Recolección de la información

La información se recolectó mediante un cuestionario diseñado y se consolidó en una base de datos en Excel^®.^ Inicialmente, se recolectó la información relacionada con la evaluación clínica y se calculó el Índice Comunitario de Fluorosis de Dean.

Para recolectar información sobre las características sociodemográficas, hábitos, dieta y conocimiento sobre fluorismo se diseñó una encuesta para que los padres o tutores de los estudiantes la contestaran por vía telefónica.

Las preguntas sobre variables sociodemográficas incluían edad, sexo, año de escolaridad, estrato socioeconómico, etnia, lugar de residencia actual, lugar de residencia antes de los ocho años, tiempo en la residencia actual y ocupación del padre. Las relacionadas con los hábitos abarcaron frecuencia de cepillado, tipo de crema dental, ingestión de crema dental, aplicación de flúor, atención odontológica en los últimos tres a doce meses, frecuencia de atención odontológica y sitio de atención odontológica. Las preguntas sobre la dieta indagaban sobre tipo de sal, marca de la sal, fuente de agua de consumo, frecuencia de consumo de legumbres, cereales, verduras, lácteos, carnes rojas, pollo, pescado, frutas, alimentos procesados o del cultivo familiar y si los padres manipulaban agroquímicos ^15^^,^^16^. Finalmente, se exploró la percepción de los padres sobre las variables de conocimiento sobre la fluorosis, las consecuencias de esta condición y si se le había diagnosticado antes al participante.

### Análisis de los datos

Los datos se analizaron con el *software* SPSS™ Statistics, versión 25 (IBM, Chicago, IL, USA). Se incluyó un análisis descriptivo en el que se calcularon proporciones, medidas de tendencia central y de dispersión; por otro lado, se hizo un análisis multivariado con modelos de regresión binomial negativa para estimar las razones de tasas (RR) ajustadas y no ajustadas, y el correspondiente intervalo de confianza del 95 % (IC _95 %_). Primero, se ejecutaron modelos de regresión binomial negativa no ajustados, para estimar la razón de tasas no ajustadas (IC _95 %_) y los valores de p para cada una de las covariables. En este paso, cualquier covariable con un valor p inferior a 0,1 era candidata para ser probada en el modelo final de regresión binomial negativa ajustado. Solo las covariables que obtuvieron un valor de p inferior a 0,05 -al ser evaluadas en simultáneo- se conservaron en el modelo final.

Para evaluar la bondad de ajuste del modelo final, se determinó la razón entre la desviación residual y los grados de libertad, y se aplicó la prueba de ji al cuadrado, usando los resultados de la desviación residual. Finalmente, se estimó el factor de inflación de la varianza para diagnosticar la multicolinealidad y se calculó la correlación entre variables.

## Resultados

En la muestra encuestada, se incluyeron 116 participantes. La edad media fue de 9,80 (DE = 1,38) años, con predominio del sexo femenino (58,6 %). El 75,9 % de los participantes vivía en la zona urbana. Las características sociodemográficas de los participantes incluidos en este estudio se presentan en el cuadro 1.

**Cuadro 1:** Características sociodemográficas de niños del municipio de La Cruz (Nariño), Colombia (N = 116)

Variables		n	%
Sexo			
	Femenino	68	58,6
	Masculino	48	41,4
Año escolar			
	Segundo	18	15,5
	Tercero	19	16,4
	Cuarto	23	19,8
	Quinto	26	22,4
	Sexto	30	25,9
Estrato socioeconómico			
	Uno	109	94
	Dos	7	6
Categoría racial			
	Blanca	55	47,4
	Mestiza	61	52,6
Lugar de residencia actual			
	Rural	28	24,1
	Urbana	88	75,9
			
Lugar de residencia anterior	Rural	29	25
	Urbana	87	75
Ocupación del padre			
	Trabajador por cuenta propia no profesional ni técnico	88	75,9
	Otros	2	24,1
**Variable**		**Media (DE)**	**Min-Max**
Edad (años)		9,80 (1,38)	8-12
Tiempo en la residencia actual (años)		9,72 (1,54)	2-12

La media del índice de Dean fue de 2,36 (DE = 0,86), que indica un compromiso muy leve en el 44,8 % de los individuos. Respecto a los hábitos relacionados con la salud oral, el 79,3 % usaba crema dental de adulto, el 91,4 % respondió que no ingería la crema dental y el 61,2 % de los participantes respondió que había asistido a consulta odontológica en los últimos 3 a 12 meses. Se encontró que el 89,7 % no tenía conocimiento sobre la fluorosis, el 95,7 % no sabía sobre las consecuencias de la enfermedad y el 94 % respondió que no se les había informado que tenían fluorosis. Las características de cuidado oral de los participantes incluidos en este estudio se presentan en el cuadro 2.

**Cuadro 2:** Cuidado oral de niños del municipio de La Cruz (Nariño), Colombia (N = 116)

Variables		n	%
Frecuencia de cepillado			
	Una o dos veces por día	55	47,4
	Tres o más veces por día	61	52,6
Tipo de crema dental			
	Adulto	92	79,3
	Niño	24	20,7
Ingestión de crema dental			
	No	106	91,4
	Sí	10	8,6
Aplicación de flúor			
	No	24	20,7
	Sí	59	50,9
	No sabe	33	28,4
Atención odontológica (últimos tres a			
	doce meses)		
	No	45	38,8
	Sí	71	61,2
Frecuencia de atención odontológica			
	Una o dos veces por año	70	60,3
	Tres o más veces por año	34	29,3
	No asiste/No sabe	12	10,3
Sitio de atención odontológica			
	EPS	95	81,9
	Particular	21	18,1
Conocimiento sobre fluorosis			
	No	104	89,7
	Sí	12	10,3
Consecuencias de la fluorosis			
	No	111	95,7
	Sí	5	4,3
¿Le han informado que tiene fluorosis?			
	No	109	94
	Sí	7	6
Gravedad de la fluorosis			
	Normal	2	1,7
	Cuestionable	13	11,2
	Muy leve	52	44,8
	Leve	39	33,6
	Moderada	10	8,6
**Variable**		**Media (DE)**	**Min-Max**
Índice de Dean		2,36 (0,86)	0-4

Respecto a la marca de sal, el 96,6 % de los participantes consumía la marca más comercial a nivel nacional; el 72,4 % refirió que la fuente de consumo de agua era “de la llave” (directamente del grifo conectado al sistema de acueducto público) y el 59,5 % refirió que consumía alimentos del cultivo familiar. También, se evaluaron hábitos alimenticios según el consumo de legumbres, cereales, verduras, lácteos, carnes rojas, pollo, pescado, frutas y alimentos procesados. Las características de los hábitos relacionados con la dieta de los participantes de este estudio, se presentan en el cuadro 3.

**Cuadro 3:** Hábitos relacionados con la alimentación de niños del municipio de La Cruz (Nariño), Colombia (N = 116)

Variables		n	%
Tipo de sal			
	Marina	6	5,2
	Refinada	110	94,8
Marca de sal			
	Refisal	112	96,6
	Otras	4	3,4
Fuente de consumo de agua			
	“De la llave”	84	72,4
	Filtrada o de botella	32	27,6
Frecuencia de consumo de legumbres			
	Dos o más veces por semana	88	75,9
	Una vez por semana	27	23,3
	Ocasionalmente o nunca	1	0,9
Frecuencia de consumo de cereales			
	Dos o más veces por semana	115	99,1
	Una vez por semana	1	0,9
Frecuencia de consumo de verduras			
	Dos o más veces por semana	48	41,4
	Una vez por semana	44	37,9
	Ocasionalmente o nunca	24	20,7
Frecuencia de consumo de lácteos			
	Dos o más veces por semana	70	60,3
	Una vez por semana	34	29,3
	Ocasionalmente o nunca	12	10,3
Frecuencia de consumo de carnes rojas			
	Dos o más veces por semana	45	38,8
	Una vez por semana	59	50,9
	Ocasionalmente o nunca	12	10,3
Frecuencia de consumo de pollo			
	Dos o más veces por semana	62	53,4
	Una vez por semana	46	39,7
	Ocasionalmente o nunca	8	6,9
Frecuencia de consumo de pescado			
	Dos o más veces por semana	4	3,4
	Una vez por semana	15	12,9
	Ocasionalmente o nunca	97	83,6
Frecuencia de consumo de frutas			
	Dos o más veces por semana	89	76,7
	Una vez por semana	21	18,1
	Ocasionalmente o nunca	6	5,2
Consumo de alimentos procesados			
	No	35	30,2
	Sí	81	69,8
Consumo del cultivo familiar			
	No	47	40,5
	Sí	69	59,5
Manipulación de agroquímicos (padres)			
	No	94	81
	Sí	22	19

En el análisis multivariado, el modelo final ajustado constó de nueve variables (lugar actual de residencia, edad, conocimientos sobre fluorosis, diagnóstico de fluorosis, marca de sal, frecuencia de consumo de legumbres, frecuencia de consumo de cereales, frecuencia de consumo de carnes rojas y frecuencia de consumo de pescado). Las personas que tenían más probabilidades de presentar un índice de Dean elevado, fueron aquellas de mayor edad (RR = 1,024; IC_95%_: 1,008-1 ,039), quienes consumían marcas de sal diferentes a la más comercial en Colombia (RR = 1,079; IC_95%_: 1,0201,142) y aquellas que informaron un mayor consumo semanal de legumbres (dos o más veces, RR = 1,118; IC_95%_: 1,045-1,197; y una vez, RR = 1,102; IC_95%_: 1,022-1. También, se encontró un mayor riesgo entre los que reportaron consumir cereales (RR = 1,080; IC_95%_: 1,003-1,164) o pescado (RR = 1,071; IC_95%_: 1,009-1,137) dos o más veces por semana, en comparación con quienes los consumían una vez por semana. Por otro lado, aquellos que informaron consumir carnes rojas dos o más veces por semana, tenían menor probabilidad de obtener un índice de Dean elevado (RR = 0,935; IC_95%_: 0,8850,988), que quienes lo hacían ocasionalmente o nunca (cuadro 4).

**Cuadro 4:** Factores asociados con la fluorosis dental (índice de Dean) en niños del municipio de La Cruz (Nariño), Colombia (N = 116)

Variable		Índice de Dean (media; mediana)	Razón de tasas no ajustadas (IC 95%)	p	Razón de tasas ajustadas (IC 95%)	p
Sexo						
	Femenino (n = 68)	2,4; 2,0	1	0,892		
	Masculino (n = 48)	2,4; 2,5	1,003 (0,963-1,044)			
Estrato socioeconómico				0,497		
	Dos (n = 7)	2,6; 3,0	1			
	Uno (n = 109)	2,4; 2,0	0,974 (0,903-1,051)			
Categoría racial				0,650		
	Mestiza (n = 61)	2,3; 2,0	1			
	Blanca (n = 55)	2,4; 2,0	1,009 (0,970-1,049)			
Lugar actual de residencia						
	Rural (n = 28)	2,6; 3,0	1	0,034	1	0,222
	Urbana (n = 88)	2,3; 2,0	0,957 (0,919-0,997)		0,974 (0,934-1,016)	
Ocupación del padre						
	Otros (n = 28)	2,4; 2,5	1	0,974		
	Trabajador por cuenta propia no profesional ni técnico (n = 88)	2,4; 2,0	1,001 (0,953-1,051)			
Edad (años)						
	(Media/DE)	9,8 (1,38)	1,029 (1,014-1,043)	0,000	1,024 (1,008-1,039)	0,003*
	(Mediana/rango)	10,0 (8-12)				
Frecuencia de cepillado						
	Tres o más veces por día (n = 61)	2,4; 2,0	1	0,527		
	Una o dos veces por día (n = 55)	2,3; 2,0	0,987 (0,949-1,027)			
Tipo de crema dental						
	Adulto (n = 92)	2,4; 2,0	1	0,653		
	Niño (n = 24)	2,3; 2,0	0,989 (0,941-1,039)			
Ingestión de crema dental						
	No (n = 106)	2,4; 2,0	1	0,602		
	Sí (n = 10)	2,2; 2,5	0,977 (0,894-1,067)			
Aplicación de flúor						
	Sí (n = 59)	2,3; 2,0	1			
	No (n = 24)	2,3; 2,0	0,988 (0,935-1,045)	0,808		
	No sabe (n = 33)	2,5; 3,0	1,018 (0,975-1,062)	0,416		
Atención odontológica (tres a doce meses)						
	Sí (n = 71)	2,4; 2,0	1	0,949		
	No (n = 45)	2,4; 2,0	0,999 (0,958-1,041)			
Frecuencia de atención odontológica						
	No asiste/No sabe (n = 12)	2,4; 2,5	1	0,892		
	Una o dos veces por año (n = 70)	2,4; 2,0	0,994 (0,918-1,078)			
	Tres o más veces por año (n = 34)	2,3; 2,0	0,988 (0,910-1,074)	0,783		
Sitio de atención odontológica						
	Particular (n = 21)	2,4; 2,0	1	0,899		
	EPS (n = 95)	2,4; 2,0	0,997 (0,954-1,042)			
Conocimientos sobre fluorosis						
	No (n = 104)	2,3; 2,0	1	0,027	1	0,667
	Sí (n = 12)	2,8; 3,0	1,059(1,006-1,115)		1,017 (0,941-1,100)	
Consecuencias de fluorosis						
	No (n = 111)	2,3; 2,0	1	0,130		
	Sí (n = 5)	2,8; 3,0	1,051 (0,985-1,122)			
¿Le han informado que tiene fluorosis?						
	No (n = 109)	2,3; 2,0	1	0,007	1	0,235
	Sí (n = 7)	3,0; 3,0	1,073 (1,020-1,129)		1,043 (0,973-1,118)	
Tipo de sal						
	Marina (n = 6)	2,2; 1,5	1	0,730		
	Refinada (n = 110)	2,4; 2,0	1,028 (0,878-1,204)			
Marca de sal						
	Refisal (n = 61)	2,3; 2,0	1	0,005	1	0,008*
	Otras (n = 96)	2,3; 3,5	1,093 (1,027-1,163)		1,079 (1,020-1,142)	
Fuente de consumo de agua						
	“Agua de la llave” (n = 84)	2,4; 2,0	1	0,723		
	Filtro o agua en botella (n = 32)	2,4; 2,0	1,008 (0,966-1,051)			
Frecuencia de consumo de legumbres						
	Nunca u ocasionalmente (n = 1)	2,0; 2,0	1	0,010	1	0.012*
	Una vez por semana (n = 27)	2,3; 2,0	1,050 (1,012-1,090)		1,102 (1,022-1,189)	
	Dos o más veces por semana (n = 88)	2,4; 2,0	1,056 (1,032-1,080)	0,000	1,118 (1,045-1,197)	0.001*
Frecuencia de consumo de cereales						
	Una vez por semana (n = 1)	2,0; 2,0	1	0,000	1	0.041*
	Dos o más veces por semana (n = 115)	2,4; 2,0	1,054 (1,034-1,075)		1,080 (1,003-1,164)	
Frecuencia de consumo de verduras						
	Nunca u ocasionalmente (n = 24)	2,3; 2,0	1	0,389		
	Una vez por semana (n = 44)	2,5; 2,0	1,026 (0,967-1,089)			
	Dos o más veces por semana (n = 48)	2,3; 2,0	1,011 (0,951-1,076)	0,726		
Frecuencia de consumo de lácteos						
	Nunca u ocasionalmente (n = 12)	2,3; 2,5	1	0,380		
	Una vez por semana (n = 34)	2,5; 3,0	1,035 (0,958- 1,118)			
	Dos o más veces por semana (n = 70)	2,3; 2,0	1,007 (0,933-1,087)	0,863		
Frecuencia de consumo de carnes rojas						
	Nunca u ocasionalmente (n = 12)	2,9; 3,0	1	0,008	1	0,078
	Una vez por semana (n = 59)	2,4; 2,0	0,947 (0,909-0,986)		0,959 (0,916-1,005)	
	Dos o más veces por semana (n = 45)	2,2; 2,0	0,920 (0,876-0,967)	0,001	0,935 (0,885-0,988)	0,017*
Frecuencia de consumo de pollo						
	Nunca u ocasionalmente (n = 8)	2,5; 2,5	1	0,554		
	Una vez por semana (n = 59)	2,4; 2,0	0,985 (0,935-1,037)			
	Dos o más veces por semana (n = 45)	2,3; 2,0	0,981 (0,935-1,029)	0,424		
Frecuencia de consumo de pescado						
	Nunca u ocasionalmente (n = 97)	2,3; 2,0	1	0,750	1	0,945
	Una vez por semana (n = 15)	2,4; 2,0	1,009 (0,956-1,065)		1,002 (0,945-1,063)	
	Dos o más veces por semana (n = 4)	3,0; 3,0	1,072 (1,008-1,140)	0,028	1,071 (1,009-1,137)	0,025*
Frecuencia de consumo de frutas						
	Nunca u ocasionalmente (n = 6)	1,8; 2,0	1	0,273		
	Una vez por semana (n = 21)	2,5; 3,0	1,101 (0,927-1,307)			
	Dos o más veces por semana (n = 89)	2,4; 2,0	1,087 (0,921-1,283)	0,324		
Consumo de alimentos procesados						
	No (n = 35)	2,4; 2,0	1	0,565		
	Sí (n = 81)	2,3; 2,0	0,988 (0,949-1,029)			
Consumo del cultivo familiar						
	No (n = 47)	2,3; 2,0	1	0,251		
	Sí (n = 69)	2,4; 2,0	1,023 (0,984- 1,064)			
Manipulación de agroquímicos por parte de los padres						
	No (n = 94)	2,3; 2,0	1	0,417		
	Sí (n = 22)	2,5; 3,0	1,021 (0,971-1,073)			

Los parámetros de bondad de ajuste del modelo fueron adecuados. La prueba de ji al cuadrado a partir de los resultados de la desviación residual resultó en un valor de p igual a 0,083 y la razón entre la desviación residual y los grados de libertad fue de 0,115, lo cual indica un buen ajuste del modelo. Los valores del factor de inflación de la varianza fueron inferiores a 10, mientras que los coeficientes de correlación fueron menores de 0,6. Estos indicadores evidencian que no hay problemas con la colinealidad.

## Discusión

Se encontró que el índice de Dean estaba asociado con algunos factores sociodemográficos, hábitos de higiene dental, dieta y conocimiento sobre la fluorosis. Los participantes con mayor probabilidad de tener un índice de Dean alto fueron aquellos de mayor edad, quienes consumían marcas de sal (diferente a la más comercial en Colombia) y aquellos con un consumo más frecuente de legumbres, cereales o pescado. En contraste, los individuos que informaron un mayor consumo de carnes rojas tenían menor probabilidad de tener un índice de Dean elevado.

Según la literatura, en los primeros ocho años de vida ocurre el periodo crítico o de exposición para el desarrollo de la enfermedad ^16^. En uno de los reportes publicados, se encontraron diferencias estadísticamente significativas entre la frecuencia elevada de fluorosis en niños de 8 a 11 años respecto a la baja frecuencia observada en los de 6 y 7 años ^17^.

Los hallazgos de este estudio evidenciaron diferencias estadísticas significativas en la gravedad de la fluorosis dental según la edad (mayor edad, mayor índice de Dean). Este resultado es lógico ya que una mayor exposición de la corona clínica -debido a la erupción dental propia del crecimiento- facilita la evaluación de posibles alteraciones dentales, lo que podría reflejar mayor gravedad de la condición.

En Colombia, en el estudio de Ramírez *et al.*, se reportó que el 60,4 % de las muestras de sal recolectadas en los hogares evaluados no cumplían con la norma establecida para la concentración de flúor. De las muestras analizadas, 10 (20,8 %) tenían más de 220 ppm y 19 (39,6 %) tenían menos de 180 ppm ^18^. No obstante, según las normas vigentes (Decreto 547 de 1996), la sal para consumo humano debe contener entre 180 y 220 ppm de flúor en su forma fluorada ^19^. Dado que las concentraciones de flúor varían incluso en lotes diferentes de una misma marca, es importante considerar su regulación entre las diferentes marcas de sal. Los resultados de este estudio evidenciaron que aquellos que consumían marcas de sal diferentes a la más comercial presentaban un mayor índice de Dean.

Salas-Zambrano reportó que las fuentes naturales con mayor contribución de flúor son el té y el pescado de mar consumido con espinas, seguidos de las carnes, los huevos, las frutas y los cereales. Sin embargo, las diferencias significativas en la ingestión de flúor observadas entre diversas comunidades se deben principalmente al agua y las bebidas, ya que los alimentos no aportan cantidades significativas a menos que en su proceso de elaboración se empleen aguas ricas en flúor ^20^. Las frutas, los vegetales, las carnes y los pescados aportan cantidades relativamente escasas de flúor. La mayoría de los vegetales y la carne contienen menos de 1 mg/kg de flúor en estado seco. En cambio, el té puede contener hasta 150 mg/kg, mientras que los pescados enlatados o ahumados y los mariscos pueden llegar a tener 20 mg/kg ^21^.

Como lo demostró el estudio de Vitoria *et al*., la ingestión diaria total de flúor depende fundamentalmente de su concentración en el agua utilizada para preparar los alimentos ^21^. Sin embargo, es lógico que la cantidad de alimentos consumida -producto de las circunstancias sociales en las cuales interactúa la población- sea clave en la ingestión de flúor, especialmente de niños y otros familiares. Entender este proceso complica la situación, ya que los problemas sociales repercuten en situaciones clínicas ^22^ que el odontólogo debe enfrentar.

Vlachou *et al.* analizaron la presencia de fluoruro en 113 muestras de alimentos y bebidas, y encontraron un amplio rango de concentraciones en productos lácteos, cárnicos, cereales, vegetales, frutas, postres y bebidas. Ninguno de los alimentos o bebidas contenía fluoruro en una concentración suficientemente grande como para ser motivo de alerta o contribuir al desarrollo de fluorosis cuando se usaba de manera convencional ^23^. Rivera *et al*. informaron que la dieta más consumida por los niños que presentan fluorosis dental fue la hipercalórica (rica en carbohidratos) e hiperproteica (rica en proteínas). Estas dietas incluyen alimentos como arroz, carnes rojas, pollo, cereales, pescado y legumbres; aunque tienen un aporte natural de flúor, la fluorosis que producen es leve ^24^. Por su parte, Aguilera *et al.* reportaron los valores de concentración promedio de fluoruro por alimento y encontraron que las sardinas tenían la mayor concentración, independientemente del lugar de recolección; a estas le siguieron los quesos, el pollo y las pastas. Las concentraciones más bajas se encontraron en la carne, los frijoles, la harina de maíz, el arroz, la leche y el aceite ^25^. Los hallazgos de este estudio demostraron que, entre más consumo de carne, menor índice de Dean. Aunque, este resultado requiere de una investigación más específica en la población, una posible explicación es que, al tratarse de una proteína que genera mayor saciedad, su consumo podría resultar en una reducción secundaria de la ingestión de otros alimentos que, directa o indirectamente, podrían estar relacionados con la fluorosis.

Otras variables relacionadas con la dieta, como el consumo frecuente de legumbres, cereales y pescado se asociaron con un mayor índice de Dean. Llama la atención que aquellos alimentos cuyo consumo requiere del riego de agua -como legumbres o vegetales - aumentaban la probabilidad de los participantes de tener un mayor índice de Dean.

La Organización Mundial de la Salud estableció que las mayores concentraciones de flúor se encuentran al pie de cadenas montañosas y en lugares delimitados geográficamente, donde el mineral ingresa en la hidrósfera, específicamente al agua subterránea, por filtración desde los suelos o los depósitos de rocas volcánicas. La concentración de flúor en el agua es variable según la región. Las aguas subterráneas son las que mayor porcentaje de fluoruros contienen, a diferencia de las superficiales. El flúor es un componente que penetra en el cuerpo humano, fundamentalmente por medio del agua y los alimentos; algunos estudios han demostrado que el agua es epidemiológicamente la fuente de flúor ^26^^,^^27^.

Los resultados del índice de Dean obtenidos en este estudio están asociados con la zona geográfica donde se encuentra ubicado el municipio de La Cruz. Es posible que el agua, especialmente la subterránea, esté relacionada con el desarrollo de fluorosis en la población evaluada. Estos individuos están expuestos constantemente a concentraciones elevadas de compuestos fluorados mediante el consumo directo del agua, la preparación de alimentos, el riego de cultivos o, simplemente, por la siembra en estos terrenos. Esta perspectiva permite abordar problemas de enfermedad desde un enfoque de salud, un aspecto clave en la salud pública del siglo XXI. La toma de decisiones debe trascender hacia la búsqueda de escenarios que entiendan holísticamente el proceso social de las poblaciones ^28^.

Existen otras formas de fluorosis dental en las que intervienen otros factores, por ejemplo, cocinar durante mucho tiempo algunos alimentos a altas temperaturas en estufas de carbón, leña o barro ^29^. Asimismo, se han reportado asociaciones entre la frecuencia de cepillado y los ingresos económicos, con la gravedad de la fluorosis dental ^30^. Sin embargo, en este estudio no se encontró asociación entre los hábitos de salud oral o el status socioeconómico, y la fluorosis.

Es necesario reconocer ciertas limitaciones inherentes a este estudio, que no se pueden controlar. Su diseño es transversal, lo que impide establecer la relación temporal y la causalidad entre el resultado, y los factores asociados. Este trabajo se basa en los resultados de una encuesta realizada a los padres o tutores de los participantes, por lo que es importante considerar el sesgo de memoria de las variables incluidas. Por último, el análisis propuesto podría haberse desarrollado con otras metodologías que, además, incluyeran el componente cualitativo, clave para futuros estudios. A pesar de las limitaciones, esta investigación tiene un amplio alcance en salud pública, ya que es el primer estudio en la región sobre fluorosis dental. Esto pone de manifiesto la necesidad de monitorear zonas del país con aumento de fluorosis dental, dadas las múltiples implicaciones que el exceso de flúor puede tener a nivel sistémico en los pacientes.

Sería útil llevar a cabo estudios longitudinales para evaluar la temporalidad y la causalidad de la exposición a compuesto fluorados ^31^. Por lo tanto, se recomienda realizar investigaciones a largo plazo que fortalezcan los hallazgos de este estudio, que evalúen otros factores asociados al desarrollo de la fluorosis dental y que involucren otras metodologías como inteligencia artificial o *machine learning*, herramientas que podrían ser clave para el análisis de problemáticas en salud oral ^32^. Además, teniendo en cuenta la alta prevalencia encontrada, es necesario ampliar la muestra para obtener resultados más contundentes. La fluorosis dental es un problema multifactorial que requiere diferentes abordajes según las particularidades de cada territorio, así como enfoques integrales de salud pública, para controlar su alta prevalencia. Esta condición plantea importantes desafíos en materia de educación, prevención y seguimiento.

## Archivos suplementarios

**Material suplementario:** Tabla epidemiológica del índice de Dean

Diente	Código	Diente	Código
17		37	
16		36	
15/55		35/75	
14/54		34/74	
13/53		33/73	
12/52		32/72	
11/51		31/71	
21/61		41/81	
22/62		42/82	
23/63		43/83	
24/64		44/84	
25/65		45/85	
26		46	
27		47	

Instrucciones de diligenciamiento:

1. Observe la presencia de fluorosis dental en cada una de las superficies vestibulares de los dientes y marque según el código que corresponda.
Nota: Si el diente no ha erupcionado en la boca, coloque un guión en la casilla.
2. Para determinar la gravedad de la fluorosis, se tendrá en cuenta el mayor nivel registrado en un diente. Primero, se diligenciará la casilla “Presencia de fluorosis dental” y, luego, con una “X”, se marcará la casilla del grado de compromiso.
3. Encierre en un círculo si el diente examinado era temporal o permanente.

**Table:**

Presencia de fluorosis dental	Normal 0	Cuestionable 1	Muy leve 2	Leve 3	Moderado 4	Grave 5
					

**Table:**

	Índice de Dean
Código	Características
0 - Normal	La superficie del esmalte es lisa, brillante y generalmente de un color blanco- crema pálido.
1 - Cuestionable	El esmalte muestra ligeras aberraciones respecto a la translucidez del esmalte en un diente sano; estas pueden fluctuar entre unas pocas manchas blancas hasta manchas ocasionales.
2 - Muy leve	Pequeñas zonas opacas de color blanco papel diseminadas irregularmente por el diente en menos del 25 % de la superficie dental vestibular
3 - Leve	Las zonas opacas blancas del esmalte extendidas en el 25 % a menos del 50 % de la superficie dental vestibular
4 - Moderado	Las superficies del esmalte de los dientes muestran un desgaste marcado y manchas carmelitas o marrones que, a menudo, se asocian con una característica desfigurante.
5 - Grave	Las superficies del esmalte están muy afectadas y la hipoplasia es tan marcada, que la forma general del diente se puede ver afectada. Existen fosas discontinuas o confluyentes. Las manchas marrones están extendidas y los dientes tienen un aspecto corrosivo.
